# Correlation of dynamic changes in γ-H2AX expression in peripheral blood lymphocytes from head and neck cancer patients with radiation-induced oral mucositis

**DOI:** 10.1186/1748-717X-8-155

**Published:** 2013-06-26

**Authors:** Ping Li, Cheng-run Du, Wen-cai Xu, Ze-liang Shi, Qing Zhang, Zhao-bin Li, Shen Fu

**Affiliations:** 1 Department of Radiation Oncology, Sixth People’s Hospital of Jiao Tong University, Shanghai 200233, People’s Republic of China

**Keywords:** γ-H2AX, Radiotherapy, Peripheral blood lymphocytes, Oral mucositis, Head and neck cancer

## Abstract

**Background:**

To evaluate the role of γ-H2AX in peripheral blood lymphocytes (PBLs) as a predictive biomarker of the severity of oral mucositis (OM) in head and neck cancer (HNC) patients with receiving radiotherapy.

**Methods:**

In vitro assays for evaluating DNA damage and repair kinetics were performed on blood samples withdrawn from 25 HNC patients undergoing radiotherapy or chemoradiotherapy before radiotherapy. As for the in vivo study, blood samples were also withdrawn before radiotherapy, and 1 hour after radiotherapy on the fourth and last days. Flow cytometry was used to assess the expression of γ-H2AX in PBLs. OM was assessed using the World Health Organization (WHO) scores twice a week and correlated with the expression of γ-H2AX.

**Results:**

The in vitro assay results showed that patients with severe OM had higher γ-H2AX-specific relative fluorescence at various irradiation doses in the damage kinetics assay, with significantly higher γ-H2AX expression at 8 Gy (*p* = 0.039), and also at 24 hours after irradiation at a dose of 2 Gy in the repair kinetics assay, compared to the patients with mild OM (*p* = 0.008). The optimal cutoff value for relative fluorescence of γ-H2AX was 0.960, 24 hours post-irradiation. However, there were no significant differences in γ-H2AX expression at different times between the two groups, as assessed with the in vivo assay.

**Conclusions:**

These results suggest that the damage and repair kinetics of γ-H2AX from PBLs in the in vitro study may have predictive value for identifying the grades of OM among HNC patients prior to radiotherapy.

## Background

Radiotherapy is one of the main therapies used for treatment of head and neck cancer (HNC). However, even with highly-refined treatment planning, such as intensity-modulated radiation therapy, radiation-induced toxicities in tumor-adjacent normal tissues remain the major limiting factor for the treatment [1]. Oral mucositis (OM) is one of the most common and severe acute normal tissue toxicities for HNC patients treated with radiotherapy [2]. OM is associated with considerable pain, dysphagia, and dysgeusia, resulting in the impairment of the quality of life. Furthermore, OM is a dose-limiting toxicity and can lead to treatment interruption, consequentially compromising the likelihood of cure [3]. There are many factors, including radiation fractionation, dose per fraction, and irradiated volume, that are associated with OM; even if nearly identical irradiation schemes are administered in the clinic, there are inter-individual heterogeneities in OM among HNC patients [4]. Therefore, an assay that enables the prediction of the grades of OM before commencing radiotherapy is highly desirable. Such predictive assays may help differentiate radiosensitive individuals from others and allow the treating physicians to modify the treatment strategies for individual patients to prevent the occurrence of severe side effects [5,6].

The extent of radiation-induced DNA damage and its repair have been considered to be the most relevant indicators of irradiation-associated toxicity to normal tissue [7]. A key factor in the repair process of damaged DNA is the histone protein H2AX, which is rapidly phosphorylated at sites of DNA double strand breaks (DSBs) and can be visualized using immunofluorescence within a few minutes of exposure of cells to DNA-damaging agents [8]. Therefore, H2AX levels have been used to quantitate the ability of cells to damage and repair DNA after irradiation [9]. The correlation of γ-H2AX with DNA damage in peripheral blood lymphocytes (PBLs), blood mononuclear cells, skin, or other human tissues has been observed [10]. However, studies of the association between the expression of γ-H2AX and the risk of normal tissue toxicities have not yielded conclusive results [11,12]. A study by Rube et al. [13] indicated that γ-H2AX of lymphocytes from children with solid tumors could be used to identify DSB-repair deficiencies in patients who developed severe toxicities to normal tissue after chemo/radiotherapy. Another study [14] also identified patients who experienced severe atypical toxicities following radiotherapy based on γ-H2AX expression analysis. However, no correlation was found by Werbrouck et al. [15] between the kinetics of the appearance of γ-H2AX foci and the severity of acute normal tissue reactions.

In this study, we measured the expression of γ-H2AX using flow cytometry in PBLs using both in vitro and in vivo assays, and investigated the pattern and kinetics of the appearance of γ-H2AX foci as predictive markers for the severity of OM in HNC patients treated with radiotherapy.

## Methods

### Patients and clinical conditions

Between November 2011 to June 2012, 31 patients with HNC treated at the Sixth People’s Hospital of Jiao Tong University were considered for enrollment in this study. The study was approved by the local ethics committee, and all patients provided written informed consent. Four patients were excluded from the study due to treatment interruptions. Two patients, who received X-ray examination or scintigraphy within 48 hours before collection of the first blood samples, were also excluded. The patients were irradiated with a 6MV X-ray linear accelerator (Artiste™ Solutions; Siemens, Germany). The mean dose of radiation was (66.2 ± 8.1) Gy, at 2 Gy per day administered 5 days per week. During radiotherapy, all patients received basic oral care.

### Assessment of OM

OM assessments were performed by a specially-trained radiation oncologist twice a week. Evaluation of OM was based on the World Health Organization (WHO) classification (1 = soreness and erythema; 2 = ulcers, can eat solids; 3 = ulcers, requires liquid diet; 4 = ulcers, can not tolerate solid or liquid diet). The maximum toxicity score was used for this study. The dose-volume histogram (DVH) of oral mucosa was extracted for each patient. The entirety of the pharyngeal constrictor and oral cavity, which is considered to play an important role in the development of acute OM, was considered as “mucosa”. Contours of the oral cavity and the muscular structures of the swallowing were assessed based on previous reports [16,17].

### Isolation of PBLs

A 10-mL sample of peripheral venous blood was withdrawn from patients and placed into EDTA vacutainers. PBLs were isolated using lymphoprep™ (AXIS-SHIELD PoC AS, Oslo, Norway) according to the manufacturer’s instructions. Briefly, whole blood was diluted with an equal volume of phosphate-buffered saline (PBS), then the 20-mL of diluted blood samples were carefully layered onto 10 mL of lymphoprep™ and centrifuged at 800 × *g* for 20 minutes. Then, lymphocytes from the interphase were isolated and incubated at 37°C in a humidified atmosphere containing 5% CO_2_.

For the in vivo assay, blood samples were obtained from patients before radiotherapy (T0), 1 hour after the fourth fraction of radiotherapy (T1), and after the last fraction of radiotherapy (T2).

Blood samples obtained before radiotherapy were used for the in vitro assay. The lymphocytes were separated and divided for different experiments. For the damage kinetics assay, lymphocytes were irradiated at doses of 0, 2, 4, 6, and 8 Gy; for the repair kinetics assay, the cells was irradiated at a dose of 2 Gy and γ-H2AX expression was examined at 0, 0.5, 4, and 24 h post-irradiation.

### γ-H2AX expression assay

The lymphocytes were fixed in 4% paraformaldehyde for 10 min and permeabilized in a 0.1% solution of Triton X-100 in PBS for 10 min. Subsequent detection of γ-H2AX expression was performed after blocking in 5% fetal bovine serum (FBS) for 30 min at room temperature, using a 1:1000 dilution of the flourescein isothiocyanate (FITC)-labeled mouse monoclonal antibody against γ-H2AX (Millipore). Following incubation with the primary antibody for 2 hours, the lymphocytes were analyzed by fluorescence-activated cell sorting (FACS) using flow cytometry and the Kaluza software package (Beckman Coulter). The fluorescence intensity in the cells post-irradiation was compared to that in untreated control cells to provide a measure of the relative fluorescence.

### Statistical analysis

Statistical analysis was performed using SPSS 13.0 software. The effect of gender, treatment regimen, tumor staging, and tumor types on OM was evaluated using the χ^2^-square test; the independent t- test was used for analysis of continuous data. The relationship between the irradiation dose and relative fluorescence of γ-H2AX in PBLs was examined by linear regression analysis. Differences were considered significant when the p-value was less than 0.05. Receiver Operating Characteristic (ROC) analysis was performed and the cutoff value was determined based on the Youden index [18], and the corresponding sensitivity, specificity, and positive and negative predictive values were calculated.

## Results

### Clinical characteristics

The clinical characteristics of the patients are shown in Table 1. There were no differences in the clinical parameters between the mild and severe OM groups. Of the 25 patients, 15 patients had mild OM and were classified as grade 1–2, while 10 patients had severe OM and were classified as grade 3–4. The percentages for patients experiencing grade 1, 2, 3, and 4 OM were 12.0%, 48.0%, 32.0%, and 8.0%, respectively.

**Table 1:** Clinical characteristics of the patients

	**WHO mucostis grade**		***p *value**
	**Grade 1–2**	**Grade 3–4**	
	**n (%)**	**n (%)**	
**Age**			
**Mean**	**51.1**	**46.2**	**0.328**
**Range**	**28-80**	**32-67**	
**Gender**			
**Male**	**8 (53.3%)**	**6 (60.0%)**	**1.000**
**Female**	**7 (46.7%)**	**4 (40.0%)**	
**Treatment regime**			
**Radiotherapy**	**5 (33.3%)**	**2 (20.0%)**	**0.659**
**Chemoradiotherapy**	**10 (66.7%)**	**8 (80.0%)**	
**Tumor staging**			
**T1**	**1 (13.3%)**	**1 (10.0%)**	**0.737**
**T2**	**5 (33.3%)**	**5 (50.0%)**	
**T3**	**3 (20.0%)**	**3 (30.0%)**	
**T4**	**2 (13.3%)**	**1 (10.0%)**	
**Tx**	**4 (20.0%)**	**0 (0.0%)**	
**Tumor types**			
**Nasopharyngeal carcinoma**	**9 (60.0%)**	**10 (100%)**	**0.330**
**Nasal cavity NHL**	**2 (13.3%)**	**0 (0.0%)**	
**Nasal cavity carcinoma**	**2 (13.3%)**	**0 (0.0%)**	
**Parotid gland carcinoma**	**1 (6.7%)**	**0 (0.0%)**	
**External auditory canal carcinoma**	**1 (6.7%)**	**0 (0.0%)**	

### Relationship between the dose-distribution parameters of oral mucosa and OM

The correlation between the grades of radiation-induced OM and DVH data were analyzed (Table 2). From the DVHs of oral mucosa, mean dose (D_mean_), maximal dose (D_max_), the percentage of mucosa in the contoured regions that received a dose equal to or higher than 15 Gy (V_15_), 30 Gy (V_30_), 40 Gy (V_40_), 50 Gy (V_50_), and 60 Gy (V_60_) were calculated. None of dose-distribution parameters of oral mucosa showed significant differences between patients with mild or severe OM (*p* >0.05).

**Table 2:** dose-distribution parameters of oral mucosa

	**Patients with mild OM (Mean ± SD)**	**Patients with severe OM (Mean ± SD)**	***p *value**
**D**_**mean**_	**(4102 ± 1183) cGy**	**(4620 ± 232) cGy**	**0.119**
**D**_**max**_	**(6697 ± 608) cGy**	**(7082 ± 293) cGy**	**0.076**
**V**_**15**_	**(90.85 ± 21.97)%**	**(99.45 ± 0.79)%**	**0.152**
**V**_**30**_	**(74.57 ± 26.74)%**	**(88.14 ± 3.79)%**	**0.072**
**V**_**40**_	**(57.45 ± 27.36)%**	**(69.48 ± 7.05)%**	**0.123**
**V**_**50**_	**(34.60 ± 19.66)%**	**(39.45 ± 9.28)%**	**0.417**
**V**_**60**_	**(14.24 ± 11.25)%**	**(17.43 ± 6.88)%**	**0.433**

### Assessment of damage kinetics using γ-H2AX expression analysis in vitro

**Figure 1:**
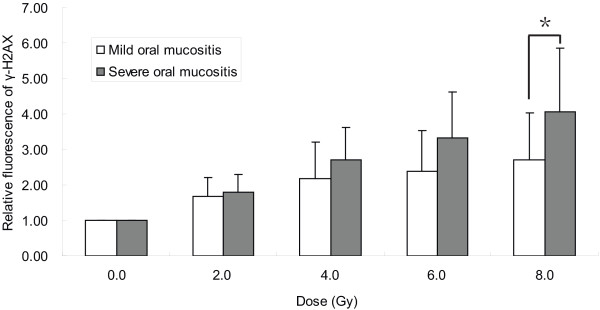
**Relationship of the applied dose of irradiation and the relative fluorescence of γ-H2AX.** Peripheral blood lymphocytes (PBLs) from patients with severe (grey bard) and mild (white bars) oral mucositis (OM) were irradiated at 0, 2, 4, 6, and 8 Gy. At 1 hour after in vitro irradiation, the γ-H2AX-associated fluorescence intensity in the lymphocytes was assessed using a FACS-based assay. The γ-H2AX-associated fluorescence is expressed relative to that of untreated control PBLs. The values shown are the mean ± SD; *p < 0.05.

After irradiation of PBLs, the fluorescence of γ-H2AX increased with the irradiation dose (Figure 1). There was a strong linear correlation between the relative fluorescence of γ-H2AX in PBLs and the irradiation dose in the patients with severe (r = 0.998; *p* = 0.000) and mild (r = 0.976; *p* = 0.005) OM. The relative fluorescence of γ-H2AX in patients with severe OM was higher than that in patients with mild OM at each irradiation dose, and was particularly high at the irradiation dose of 8 Gy (*p* = 0.039).

### Assessment of repair kinetics using in vitro γ-H2AX expression analysis

**Figure 2:**
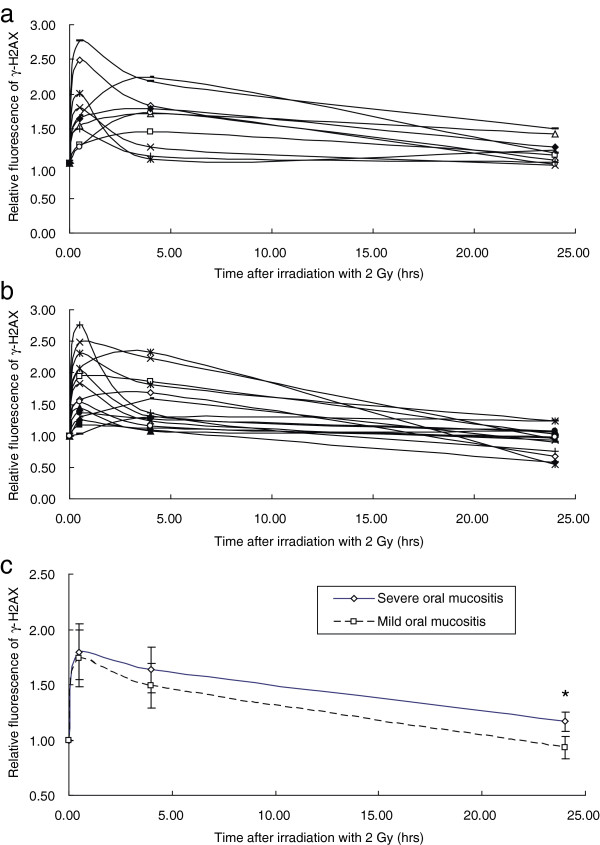
**The time-dependent accumulation of irradiation-induced γ-H2AX in PBLs in vitro.** The γ-H2AX fluorescence was measured using a FACS-based assay in PBLs isolated from 15 patients mild OM **(a)** and 10 patients with severe OM **(b)** at 0, 0.5, 4, and 24 hours after in vitro exposure to 2 Gy of X-ray radiation. **(c)** The mean level of the relative fluorescence for patients with severe OM (diamonds) and mild OM (squares); *p <0.05.

As shown in Figure 2, the relative fluorescence intensity reached the maximum level at about 0.5 h after irradiation and decreased thereafter. At 24 hours, the relative fluorescence returned to levels comparable to that of un-irradiated PBLs in mild OM patients (Figure 2a), but not in patients with severe OM, indicating a slower rate of DNA repair in patients with severe OM (Figure 2b). Furthermore, there was a significantly higher retention of fluorescence at 24 hours post-irradiation in PBLs from the severe OM group, compared to the residual fluorescence in the mild OM group (*p* = 0.008) (Figure 2c), while no difference was observed at 0.5 and 4 hours post-irradiation (Figure 2c). These results suggest that higher residual DNA damage is present in patients with severe OM 24 hours after irradiation.

### Cutoff value of γ-H2AX fluorescence for prediction of OM

**Figure 3:**
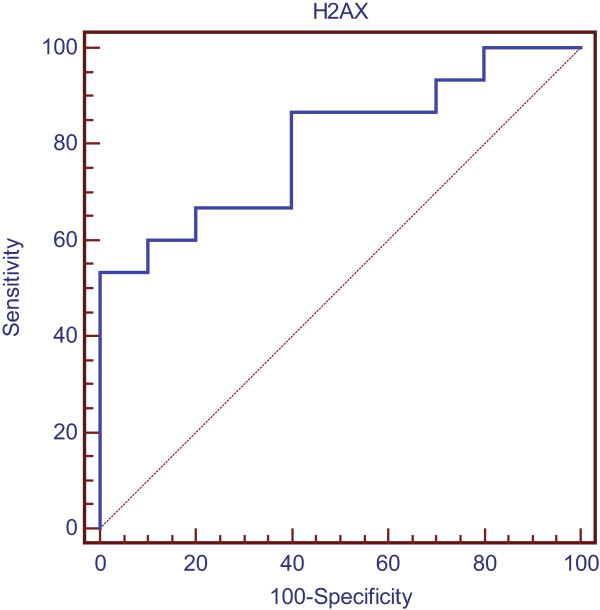
**ROC curve of residual γ-H2AX fluorescence 24 h post-irradiation for determining the grades of OM.** Receiver Operating Characteristic (ROC) analysis was performed by SPSS software and cutoff value was determined based on Youden index. The ordinate represents the true positive rate, the abscissa represents the false positive rate. The area under the curve (AUC) was used to assess the predictive value of γ-H2AX.

To estimate the sensitivity and specificity of the relative fluorescence of γ-H2AX at 24 hours post-irradiation for prediction of OM during radiotherapy, ROC analysis was performed. The optimal cutoff value of residual γ-H2AX level 24 hours post-irradiation was determined from the ROC analysis (Figure 3). When a relative fluorescence of 0.960 was used as the cutoff value, the sensitivity and specificity of the γ-H2AX fluorescence in predicting OM were 100% and 53.3%, respectively, and positive and negative predictive values were 62.5% and 100%, respectively. The area under the curve (AUC) was 0.800 (standard error [SE], 0.094; 95% confidence interval [CI], 0.593–0.931; *p* = 0.002).

### The dynamic change of γ-H2AX expression using the in vivo assay

**Figure 4:**
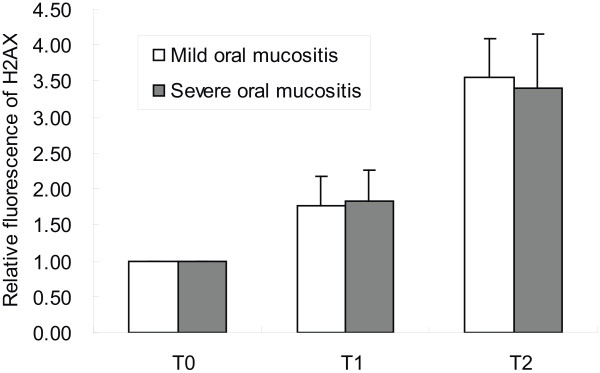
**The relationship of relative fluorescence of γ-H2AX and OM response to radiotherapy in vivo.** Blood samples were collected from patients before radiotherapy (T0), 1 hour after the fourth fraction of radiotherapy (T1), and 1 hour after the last fraction of radiotherapy (T2) and the induction of γ-H2AX was assessed using a FACS-based assay.

To determine the correlation between the kinetic patterns of DNA damage and repair, as assessed using the γ-H2AX-specific fluorescence with the in vitro assay, and OM, the relative fluorescence of γ-H2AX was estimated in 15 patients with mild OM and 10 patients with severe OM at different time points during radiotherapy (Figure 4). Higher γ-H2AX levels were observed at the later time points of radiotherapy (*p* <0.05), but the increase in γ-H2AX expression was not statistically different between the two groups of patients at the three time points during radiotherapy (Figure 4, *p* <0.05).

## Discussion

Development of severe radiation-induced OM during radiotherapy for patients with HNC results in treatment interruptions, hospitalization, and increased cost. Screening tests that can identify the patients at higher risk for developing severe OM would enable clinicians to individualize radiotherapy protocols.

The appearance of γ-H2AX foci in PBLs was found to be closely correlated with DNA DSBs after exposure to irradiation [6], and more rapid loss of γ-H2AX foci in human cell lines has been correlated with improved repair of DNA DSBs [10]. In the present study, flow cytometric analysis of γ-H2AX induction and removal was used to predict acute radiation-induced OM in HNC patients. The extent and kinetics of γ-H2AX expression levels were measured after in vitro irradiation of patient-derived PBLs, and the results of the in vitro assays were also compared with those of the in vivo analysis.

We found a strong linear correlation between the relative fluorescence of γ-H2AX in PBLs and irradiation dose in the patients with severe and mild OM, indicating that γ-H2AX might be useful as a biological marker of dosimetry after ionizing radiation exposure, which corroborates results in previous reports [19,20]. On the other hand, our data showed that the dose per fractionation might be a key parameter that can be applied to estimate the role of the induction of γ-H2AX in predicting OM in HNC patients. In the present study, the induction of γ-H2AX by high-dose irradiation (8 Gy), but not other lower doses of irradiation (2, 4, or 6 Gy), was significantly different between the patients with severe and mild OM. These data suggest that the lack of support for a predictive role for γ-H2AX in OM reported previously may be due to the lower doses of irradiation used in prior study [15]. Werbrouck et al. [15] measured the number of γ-H2AX foci in T-lymphocytes isolated from HNC patients after in vitro irradiation at 0.5 Gy and were unable to predict the development of acute normal tissue complications. An irradiation dose of 2 Gy was used in the study by Vasireddy et al. [21], which suggested that the formation of γ-H2AX foci was not significantly different between patients with severe and mild OM. Taken together, our data suggest that the patients with severe OM had higher radiosensitivity of normal tissue than those with mild OM, and the higher single dose might be necessary in in vitro assays to evaluate the correlation between γ-H2AX induction and OM in the clinical setting.

The results of the repair kinetics assay showed that lymphocytes had a higher residual level of fluorescence of γ-H2AX at 24 h, but not at earlier times ( 0, 0.5, and 4 h), after irradiation with 2 Gy in patients with severe OM, compared with patients with mild OM. Our observations are consistent with those of other studies; Bourton et al. [14] examined γ-H2AX induction and depletion in 12 patients with acute or late normal tissue toxicity using the same technique used in this study and found elevated levels of γ-H2AX fluorescence at 24 h post-irradiation in patients with severe toxicities, compared with those who had experienced little or no adverse reaction. Results from the study by Goutham et al. [22] also suggested that lymphocytes from the patients before radiotherapy had a higher residual level of fluorescence of γ-H2AX at later times (24 h) after in vitro irradiation with 2 Gy, more so in patients who developed severe OM. These results indicate that patients with severe OM are more likely to exhibit DNA repair deficiencies. Therapy-related factors that may have an important bearing on the development of mucositis, such as the use of chemotherapy and the irradiation dose to the oral cavity, were not taken into account as confounding factors in these studies [14,22]. In the present study, the related parameters from DVH with respect to oral cavity and mucosa, including D_max_, D_mean_, and V_30_, were measured and found to be similar between the patients with severe and mild OM (p >0.05); therefore, the differences between the dose to and volume of the oral mucosa did not account for the differences between the patients with severe and mild OM. Moreover, despite the different proportion of the patients with severe (8/10) and mild (9/15) OM undergoing chemoradiotherapy, there were no differences in treatment regime between patients with mild and severe OM (*p* = 0.402). Taken together, these confounding factors (concurrent chemo, dose to, and volume of the oral mucosa) were eliminated. However, the absence of the influence of concurrent chemotherapy, dose to, and volume of the oral mucosa on OM in our study may be due to the small sample size, a limitation of this study. In our analyses, the level of γ-H2AX-associated fluorescence at 24 h post-irradiation was a major contributor to the differences in the severity of acute OM. Therefore, along with that of other reports, our data indicated that the patients with severe OM had a reduced capacity for DNA repair.

ROC analysis was further used to confirm the predictive value of γ-H2AX expression, and the relative fluorescence intensity of γ-H2AX (0.965) at 24 h post-irradiation was the optimal cutoff value, with an AUC value close to 0.8 and sensitivity, specificity, and positive and negative predictive values being 100%, 53.8%, 62.5%, and 100%, respectively. These data indicate that the H2AX expression-based method used for predicting OM had fair coverage of the whole cohort and that HNC patients with relative fluorescence intensities <0.96 after in vitro irradiation would be highly likely to experience mild mucositis. The relatively lower value for the specificity and positive predictive value with the γ-H2AX expression-based approach may be a result of pharmaceutical modifiers and the lower irradiation doses applied to two of the patients (with non-hodgkin’s lymphoma) in our study.

To our knowledge, there are no previous reports of clinical data focused on the detection and predictive role of γ-H2AX in radiotherapy-induced OM, using both in vivo and in vitro assays. Measurement of the accumulation of DNA damage with the γ-H2AX-based assay has been demonstrated to enable identification of minor impairments in the DSB-repair capacity, which could not be detected after single-dose irradiation in a preclinical murine model [23]. In our study, although higher levels of γ-H2AX were observed when the patients received a higher dose of irradiation, indicating that the γ-H2AX assay reflected the accumulation of incomplete repair of sublethal damage, the enhancements of γ-H2AX expression on the fourth and last days after fractionated irradiation were not statistically different between the patients with mild and severe OM. The lack of differences between the two groups should not be seen as a failing of the role of DNA damage accumulation, since there are many factors involved in the in vivo analysis of γ-H2AX expression, such as the mixture of irradiated and non-irradiated blood lymphocytes in the blood samples, which may confound the analysis. Other complicating factors include the continuous exchange of lymphocytes between blood and tissues and elimination of highly damaged lymphocytes from blood [24]. Our results prove that the detection of γ-H2AX accumulation induced by irradiation can be used to predict the incidence and severity of normal tissue toxicities like OM. We propose that the in vitro analysis needs to be performed before radiotherapy with higher doses and over a longer observation period. In conclusion, our data support the further development of the H2AX-based method as a rapid and convenient to identify patients with higher risk of developing severe OM during radiotherapy.

Although we have observed a correlation between γ-H2AX induction and the grade of OM prior to radiotherapy, other studies have not demonstrated such a relationship [15,25]. The discrepancies in results among studies may be primarily due to technical differences in the methodologies implemented, the endpoints, and different clinicopathological characteristics of the study populations. Immunoflurorescence-based microscopic analysis of γ-H2AX is one of the methods for detection of DNA damage after irradiation. However, manual counting used in such microscopic analysis may be less accurate, especially in samples with a greater number of foci, which overlap or coalescence, such as those observed at dose ranges >2 Gy [26]. Flow cytometry is an objective and rapid method for the detection of γ-H2AX signal intensity [14]. These qualities make flow cytometry more attractive for use in the detection and quantification of γ-H2AX intensity in clinical samples.

In addition to the application of γ-H2AX-based approaches for the prediction of acute normal tissue damage, some authors also investigated the role of the γ-H2AX in the prediction of late normal tissue toxicities. However, those studies failed to demonstrate a relationship between the number or intensity of γ-H2AX foci and late radiation sensitivity [27,28]. The lack of correlation may be explained by the fact that other factors, in addition to DNA repair mechanisms, such as fibrotic and inflammatory processes, play an important role in the induction of late adverse effects.

## Conclusion

In conclusion, our data demonstrate that the H2AX-based method is suitable for application in a clinical setting. Flow cytometric analysis of γ-H2AX fluorescence allowed assessment of DNA damage and DSB repair capacity in individual HNC patients after irradiation. The dynamic changes in γ-H2AX fluorescence were evident at higher doses of irradiation and over longer periods of time after in vitro irradiation. However, the limited number of patients is a significant limitation of our study, and these observation need to be extended and confirmed in a larger cohort to thoroughly address the utility of the H2AX expression-based approach in predicting the severity of OM in HNC patients undergoing radiotherapy.

## Abbreviations

OM: Oral mucositis; HNC: Head and neck cancer; DSBs: DNA double strand breads; PBL: Peripheral blood lymphocytes; DVH: Dose-volume histogram; PBS: Phosphate-buffered saline; ROC: Receiver Operating Characteristic; AUC: The area under the curve.

## Competing interests

The authors declare that they have no competing interests in this study.

## Authors’ contributions

PL and SF designed the study. PL, CD, QZ and ZL aided in data collection. PL and CD performed the statistical analysis and draft the manuscript. WX, PL and ZS performed the γ-H2AX analysis. All authors read and approved the final manuscript.
